# Consistent Thermo-Capillarity and Thermal Boundary Conditions for Single-Phase Smoothed Particle Hydrodynamics

**DOI:** 10.3390/ma14164530

**Published:** 2021-08-12

**Authors:** Claas Bierwisch

**Affiliations:** Fraunhofer IWM, Wöhlerstr. 11, 79108 Freiburg, Germany; claas.bierwisch@iwm.fraunhofer.de; Tel.: +49-761-5142-347

**Keywords:** continuum surface force, contact angle, wetting, Marangoni flow, heat transfer, heat flux, radiation, coalescence

## Abstract

A model for capillary phenomena including temperature-dependency and thermal boundary conditions is presented in the numerical framework of smoothed particle hydrodynamics (SPH). The model requires only a single fluid phase and is therefore computationally more efficient than surface tension schemes which need an explicit fluid-fluid or fluid-gas interface. The model makes use of a surface identification mechanism based on the SPH renormalization tensor. All relevant properties of the continuum surface force (CSF) based approach, i.e., the delta function, normal vector and curvature, are calculated in a consistent manner. The model is parametrized by physical material properties and is successfully validated by means of a large set of analytical test cases. The applicability of the proposed model to more complex scenarios is demonstrated.

## 1. Introduction

Modeling surface tension in the framework of smoothed particle hydrodynamics [1,2] is generally done using one of the following two approaches.

On the one hand, surface tension can be formulated as a continuum surface force. This approach introduces a surface delta function to approximate the tension, i.e., a force per area, in form of a force per volume [3]. A color function is used here to identify the involved phase and the interface between them. An early SPH implementation of the CSF approach for two phases was described and analyzed by Morris [4]. Hu and Adams [5] used an interface stress tensor to avoid the explicit calculation of the curvature at the phase interface. They also extended the formulation to more than two phase which allows modeling of contact angles. Adami et al. [6] improved the CSF scheme by an accurate curvature calculation. Their method is also able to handle large density ratios of the involved phases of up to 1000. Zhang [7] enabled surface tension modeling in a single-phase SPH simulation by identifying the boundary particles and reconstructing the phase surface in two dimensions (2D) or three dimensions (3D). Breinlinger et al. [8] were able to model wetting effects in two-phase SPH simulations by prescribing the contact angle with respect to a rigid substrate. Thermo-capillary effects based on a temperature-dependent surface tension were studied by Tong and Browne [9] using a two-phase SPH approach. For this purpose, they used a projection of the gradient operator onto the phase interface. Yeganehdoust et al. [10] developed a method to extrapolate the color function into a substrate in order to model both static and dynamic wetting phenomena. Huber et al. [11] introduced a balance equation for the contact line between the two fluid phases and the substrate to accurately model advancing and receding contact angles in dynamic wetting situations. Ordoubadi et al. [12] used a single-phase SPH formulation for the CSF approach in which they identified the surface by a combination of a geometric interface tracking and the calculation of the position divergence field [13]. Hirschler et al. [14] formulated a single-phase model which obtains the surface normal and the surface delta function directly from the SPH kernel gradient. The surface curvature is obtained using a corrected kernel gradient [15]. Krimi et al. [16] used an interface stress tensor formulation to model multi-phase flow situations with large density ratios of the involved phases. Hopp-Hirschler et al. [17] investigated thermo-capillary phenomena in internal flow situations or for two-phase systems, respectively. They stabilized the SPH simulations by a particle shifting technique [18]. Lin et al. [19] proposed the detection of an interface between two phases as well as the calculations of the interface normal and curvature based on a Delaunay triangulation. Fürstenau et al. [20] formulated a single-phase description using the position divergence to identify the surface and SPH kernel gradient to compute the surface normal. They improved the surface curvature calculation by using a curvature tensor. Liu et al. [21] proposed a machine learning approach in order to obtain a more accurate local interface curvature in two-phase simulations. Härdi et al. [22] investigated wetting phenomena of thin films by using an SPH discretization of the shallow water equation and introducing a contact line force. Liu et al. [23] proposed a single-phase surface tension model based on the $\delta^{+}$ SPH scheme [24]. They calculated the surface normal from the kernel gradient and used a normalization procedure to improve the accuracy of the surface delta function.

On the other hand, a surface tension formulation can be based on an additional pairwise force which is repulsive at short range, attractive at intermediate range and vanishing at long range. Such models were developed by Nugent and Posch [25] and refined by Tartakovsky and Meakin [26] as well as Akinci et al. [27]. A pairwise force mimics the molecular origin of surface tension [28] and results in a net force towards the bulk for particles at a curved, convex surface [29]. The pairwise force approach originally required calibration by adjusting model parameters which have no correspondence in experiments. Tartakovsky and Panchenko [30] made inroads to overcome this drawback by deriving relations between the pairwise model parameters and physical properties for multi-phase systems. Nair and Pöschel [31] extended this approach to single-phase simulations. Bao et al. [32] improved the pairwise force formulation for dynamic wetting phenomena by introducing an apparent viscosity at the interface to a rigid substrate which mimics the effect of a finite slip length.

In this work a CSF single-phase surface tension scheme is presented which is an extension of an earlier work by the author [33]. The approach for modeling wetting behavior is adapted from Breinlinger et al. [8] and the method to incorporate thermo-capillarity from Tong and Browne [9]. The novelty of the present work is the consistent formulation which is used to compute all surface related properties. The surface identification scheme from Marrone et al. [34], relying on the renormalization tensor, is used as the basis to obtain the surface normal, surface curvature and surface delta function in a consistent and accurate manner. In previous works, at least one of these three surface properties was not based on the renormalization tensor, leading to an inconsistency in the definition of the actual surface. The surface delta function is also used to formulate thermal boundary conditions which take into account heat transfer, heat flux and radiation.

The remaining part of this paper is organized as follows. Section 2 outlines the governing equations and presents all relevant ingredients of the numerical model. Section 3 uses several benchmark cases to assess the predictive quality of the model. The obtained results are discussed in Section 4. Section 5 concludes the main part of the paper. Details of the surface delta function computation are given in Appendix A.

## 2. Materials and Methods

The governing equations with respect to mass balance, momentum balance and energy balance are presented in Section 2.1 with focus on the surface related terms. The discretization of the governing equations using SPH is described in detail in Section 2.2. The main contribution of the present work is the SPH surface treatment presented in Section 2.2.2. It forms the basis for modeling surface thermal boundary conditions (Section 2.2.4), surface tension (Section 2.2.10) and surface wetting (Section 2.2.11).

### 2.1. Governing Equations

The evolution of density ρ over time *t* is given by the mass balance equation,

(1) $$\frac{D\rho }{Dt}=-\rho \;\nabla \;\cdot \;v,$$

where v is the velocity.

The Navier–Stokes momentum equation provides the time evolution of velocity,

(2) $$\begin{array}{cc} \frac{Dv}{Dt} & \;=\;-\frac{1}{\rho }\nabla P+\frac{2\;\eta }{\rho }\nabla \;\cdot \;E+\frac{1}{\rho }F^{S}+g\\ \; & \;=\;-\frac{1}{\rho }\nabla P+\frac{\eta }{\rho }\nabla^{2}v+\frac{1}{\rho }F^{S}+g, \end{array}$$

with pressure *P*, dynamic viscosity η, rate of strain tensor $E=(\nabla v+{\left(\nabla v\right)}^{T})/2$, volumetric surface tension force $F^{S}$ and gravitational acceleration g. The time evolution of the position r is simply

(3) $$\frac{Dr}{Dt}=v.$$

The surface tension force can be decomposed into components normal and tangential, respectively, to the surface,

(4) $$F^{S}=F^{n}+F^{t}.$$

The CSF model converts a force per area, i.e., pressure or tension, into a force per volume by multiplication with a delta function $\delta_{S}$ which marks the location of the surface in space. This approach yields the following expression for the normal component of the volumetric surface tension force,

(5) $$F^{n}=-\sigma_{n}\;\kappa \;\delta_{S}\;n$$

with $\sigma_{n}$ as the surface tension, κ as the curvature and n as the surface unit normal vector. A corresponding tangential volumetric force caused by a gradient of the surface tension is given by

(6) $$F^{t}=\delta_{S}\;\nabla_{S}\;\sigma_{n}$$

with $\nabla_{S}$ as the Nabla operator along the surface. One reason for a non-constant surface tension is a dependency on temperature *T* which might be expressed as $\nabla_{S}\;\sigma_{n}=\sigma_{t}\nabla_{S}\;T$ with $\sigma_{t}=\partial \sigma_{n}/\partial T$ which is typically negative and causes Marangoni flow along the surface from warm towards cold regions. The delta function is constructed as the magnitude of the gradient of a color function which is 1 on one side of the surface (or interface) and 0 on the other side.

The evolution for the thermal energy per unit mass *H* is given by

(7) $$\frac{DH}{Dt}=\frac{k}{\rho }\nabla^{2}T+\frac{q}{\rho }\;\delta_{S}-\frac{f}{\rho }\;\delta_{S}\left(T-T_{a}\right)-\frac{\varepsilon }{\rho }\;\sigma_{B}\;\delta_{S}\left(T^{4}-T_{a}^{4}\right).$$

Here, *k* is the thermal conductivity, *q* is the heat flux, *f* is the heat transfer coefficient, ϵ is the emissivity, $\sigma_{B}$ is the Stefan–Boltzmann constant and $T_{a}$ is the ambient temperature.

The heat capacity *c* relates the temperature to the specific thermal energy,

(8) $$T=\frac{H}{c}.$$

### 2.2. SPH Discretization

#### 2.2.1. SPH Kernel Approximation

A quantity *A* at the position r is interpolated using a weighted sum over the contributions $A(r_{j})$ of all neighboring particles *j* at positions $r_{j}$ with masses $m_{j}$ and densities $\rho_{j}$,

(9) $$\langle A\left(r\right)\rangle =\sum_{j}\frac{m_{j}}{\rho_{j}}A\left(r_{j}\right)W(|r-r_{j}\left|,h\right),$$

where W(r,h) is a kernel function. The particle mass is related to the rest density $\rho_{0}$ and the initial particle spacing Δx via $m_{j}=\rho_{0}{\left(\Delta x\right)}^{d}$ where *d* is the number of spatial dimensions.

(10) $$W\left(r,h\right)=\frac{\nu }{h^{d}}\left\{\begin{array}{cc} {\left(1-\frac{1}{2}\zeta \right)}^{4}\;\left(2\;\zeta +1\right), & 0\leq \zeta <2,\\ 0, & \zeta \geq 2 \end{array}\right.$$

is the quintic C2 Wendland kernel [35] with *h* as smoothing length and ζ=r/h. The normalization factor is ν=7/(4π) for d=2 and ν=21/(16π) for d=3.

Equation (9) can be used to obtain a smoothed approximation of a field quantity at the position of particle *i* via

(11) $$\langle A_{i}\rangle =\sum_{j}\frac{m_{j}}{\rho_{j}}A_{j}W_{ij},$$

where $A_{j}=A\left(r_{j}\right)$, $W_{ij}=W(|r_{ij}\left|,h\right)$, and $r_{ij}=r_{i}-r_{j}$.

In order to discretize the governing equations in the SPH scheme, spatial gradients of field quantities can analogously be expressed via

(12) $$\nabla A_{i}=\sum_{j}\frac{m_{j}}{\rho_{j}}A_{j}\nabla W_{ij},$$

where the kernel gradient $\nabla W_{ij}$ is explicitly given by

(13) $$\nabla W_{ij}=\frac{\nu }{h^{d+1}}\frac{r_{ij}}{|r_{ij}|}\left\{\begin{array}{cc} 5\;\zeta \;{\left(\zeta -2\right)}^{3}/8, & 0\leq \zeta <2,\\ 0, & \zeta \geq 2. \end{array}\right.$$

In comparison to the canonical formulation given by Equation (12), the expressions

(14) $$\nabla A_{i}=\sum_{j}\frac{m_{j}}{\rho_{j}}\left(A_{j}-A_{i}\right)\nabla W_{ij}$$

and

(15) $$\nabla A_{i}=\rho_{i}\sum_{j}m_{j}\left(\frac{A_{i}}{\rho_{i}^{2}}+\frac{A_{j}}{\rho_{j}^{2}}\right)\nabla W_{ij}$$

prove to be numerically favorable [36] and are therefore used in the following SPH discretizations.

#### 2.2.2. SPH Surface Modeling

A discretization of the governing equations requires the computation of several surface related quantities such as the surface delta function $\delta_{S}$, the surface unit normal n, the surface curvature κ and the gradient operating along the surface $\nabla_{S}$. The following section describes our approach to obtain these quantities.

The present SPH formulation makes use of several aspects introduced in [14,24,34]. A central feature is given by the method to identify the surface by means of the renormalization tensor L. The inverse renormalization tensor for particle *i* is given by

(16) $$L_{i}^{-1}=\sum_{j}\frac{m_{j}}{\rho_{j}}\;\nabla W_{ij}⊗r_{ji}.$$

The minimum eigenvalue $\lambda_{i}$ of $L_{i}^{-1}$ defines a scalar field which is 1 inside the volume and approaches 0 at the surface of the particle distribution [34]. This property makes the λ field similar to the color function on which the CSF model is based. This analogy is used as key ingredient of the presented surface tension model. The corrected gradient of the λ field for particle *i* is given by

(17) $$\nabla \lambda_{i}=\sum_{j}\frac{m_{j}}{\rho_{j}}\;\left(\lambda_{j}-\lambda_{i}\right)\;L_{i}\;\nabla W_{ij}$$

and the surface unit normal vector follows simply as

(18) $$n_{i}=-\frac{\nabla \lambda_{i}}{|\nabla \lambda_{i}|}.$$

Following [24] we identify those particles with $\lambda_{i}\leq 0.2$ as free surface particles. For each particle *i* with $\lambda_{i}>0.2$ and $\lambda_{i}\leq 0.75$ an umbrella-shaped region with cone half angle $45^{\circ }$ and range 2Δx in the direction of the normal $n_{i}$ is scanned. If there is no particle *j* within this umbrella-shaped region, the particle *i* is also identified as free surface particle. We define the set of particles which are at most a distance of 1.5Δx away from the free surface particles as surface region particles, including the free surface particles. Thus, the surface region is typically composed of two neighboring layers of particles. In order to avoid numerical problems caused by isolated particles, we exclude particles with $\lambda_{i}<0.1$ from the surface region. All further surface related calculations are carried out only for the surface region particles defined in this paragraph.

Furthermore, we define the surface delta function by

(19) $$\delta_{i}=\Xi_{\lambda }\;\left|\nabla \lambda_{i}\right|,$$

where $\Xi_{\lambda }$ is a dimensionless normalization factor which depends on the ratio h/Δx and the actual function used as SPH kernel (see Appendix A). An alternative approach to obtain the surface delta function follows [14],

(20) $$\delta_{i}=\Xi_{1}\;|\sum_{j}\frac{m_{j}}{\rho_{j}}\;\nabla W_{ij}|=\Xi_{1}\;\left|\nabla 1_{i}\right|,$$

where $\nabla 1_{i}$ is Equation (12) applied to unity at the position of each particle *j*, i.e., $A_{j}=1$. Differences between the approaches given by Equations (19) and (20) are discussed in Appendix A.

The local curvature of the surface is then calculated following [14] using a corrected kernel gradient,

(21) $$\kappa_{i}=\nabla \;\cdot \;n_{i}=\sum_{j}\frac{m_{j}}{\rho_{j}}\;\left(n_{j}-n_{i}\right)\;\;\cdot \;\left(L_{i}\;\nabla W_{ij}\right).$$

The sum in Equation (21) is restricted to the surface region particles. The curvature is limited by the spatial resolution,

(22) $$2\;h\;|\kappa_{i}|\leq 1.$$

#### 2.2.3. SPH Mass Balance

The mass balance (Equation (1)) is discretized using Equation (14) in the form

(23) $$\frac{D\rho_{i}}{Dt}=\rho_{i}\sum_{j}\frac{m_{j}}{\rho_{j}}v_{ij}\;\cdot \;\nabla W_{ij},$$

where $v_{ij}=v_{i}-v_{j}$ is the relative particle velocity.

#### 2.2.4. SPH Thermal Energy Balance

The thermal energy balance (Equation (7)) for each SPH particle *i* takes the form

(24) $$\frac{DH_{i}}{Dt}=\left(\frac{2\;k}{\rho_{i}}\sum_{j}\frac{m_{j}}{\rho_{j}}\left(T_{i}-T_{j}\right)\frac{r_{ij}\;\cdot \;\nabla W_{ij}}{|r_{ij}{|}^{2}+\frac{1}{100}h^{2}}\right)+\frac{\delta_{i}}{\rho_{i}}\left(q-f\;\left(T_{i}-T_{a}\right)-\varepsilon \;\sigma_{B}\;\left(T_{i}^{4}-T_{a}^{4}\right)\right).$$

The first term on the right hand side representing thermal diffusion is calculated using the approach from [37]. The next three terms for heat flux, heat transfer and radiation, respectively, make use of the surface delta function given by Equation (19). The temperature for each particle *i* is then simply obtained as $T_{i}=H_{i}/c$.

#### 2.2.5. SPH Temperature Gradient

In order to model thermo-capillary effects, we need to obtain the temperature gradient within our SPH formulation similarly to [9]. A corrected gradient formulation [15] is used in order to get reliable values at the surface,

(25) $$\nabla T_{i}=\sum_{j}\frac{m_{j}}{\rho_{j}}\;\left(T_{j}-T_{i}\right)\;L_{i}\;\nabla W_{ij}.$$

#### 2.2.6. SPH Momentum Balance

The Navier–Stokes momentum balance (Equation (2)) for each SPH particle *i* takes the form

(26) $$\frac{Dv_{i}}{Dt}=f_{i}^{P}+f_{i}^{V}+f_{i}^{A}+f_{i}^{S}+g,$$

with the accelerations caused by pressure, $f_{i}^{P}$, by viscosity, $f_{i}^{V}$, by artificial viscosity, $f_{i}^{A}$ and by surface tension, $f_{i}^{S}$.

#### 2.2.7. SPH Pressure

The pressure acceleration [1] is obtained by using the gradient discretization according to Equation (15),

(27) $$f_{i}^{P}=-\sum_{j}m_{j}\left(\frac{P_{i}}{\rho_{i}^{2}}+\frac{P_{j}}{\rho_{j}^{2}}\right)\nabla W_{ij}.$$

The pressure $P_{i}$ of particle *i* is related to its density $\rho_{i}$ by Tait’s equation of state,

(28) $$P_{i}=\frac{s^{2}\rho_{0}}{\gamma }\left[{\left(\frac{\rho_{i}}{\rho_{0}}\right)}^{\gamma }-1\right],$$

where *s* is the numerical speed of sound and γ is the dimensionless isentropic exponent.

#### 2.2.8. SPH Viscosity

Depending on the details of the actual simulation scenario, one of two approaches to model the effect of viscosity on the flow is used. Cleary [37] introduced a formulation based on the relative normal velocity of each pair or particles *i* and *j*,

(29) $$f_{i}^{V}=\frac{2(2+d)\;\eta }{\rho_{i}}\sum_{j}\frac{m_{j}}{\rho_{j}}\;\frac{v_{ij}\;\cdot \;r_{ij}}{|r_{ij}{|}^{2}+\frac{1}{100}h^{2}}\nabla W_{ij}.$$

An alternative formulation is given by Sigalotti et al. [38] which is structurally similar to the pressure term (Equation (27)),

(30) $$f_{i}^{V}=2\;\eta \sum_{j}m_{j}\left(\frac{E_{i}}{\rho_{i}^{2}}+\frac{E_{j}}{\rho_{j}^{2}}\right)\nabla W_{ij}.$$

The rate of strain tensor $E_{i}=\left(\nabla v_{i}+{\left(\nabla v_{i}\right)}^{T}\right)/2$ of particle *i* is based on its velocity gradient tensor,

(31) $$\nabla v_{i}=\sum_{j}\frac{m_{j}}{\rho_{j}}v_{ji}⊗\nabla W_{ij}.$$

#### 2.2.9. SPH Artificial Viscosity

An artificial viscosity formulation is in some situations required to stabilize the numerical simulation by damping high frequency noise. It should not influence the dynamics of the simulation to a statistically significant extent. The artificial viscosity term is given by Monaghan [1],

(32) $$f_{i}^{A}=\sum_{j}m_{j}\;\Pi_{ij}^{A}\;\nabla W_{ij},$$

where

(33) $$\Pi_{ij}^{A}=\left\{\begin{array}{cc} 2\left(\alpha \;s-\beta \;\phi_{ij}\right)\phi_{ij}/\left(\rho_{i}+\rho_{j}\right), & v_{ij}\;\cdot \;r_{ij}<0,\\ 0, & v_{ij}\;\cdot \;r_{ij}\geq 0, \end{array}\right.$$

with α and β as dimensionless control parameters of the order of $10^{-2}$ and $\phi_{ij}$ as a rescaled normal velocity,

(34) $$\phi_{ij}=\frac{h\left(v_{ij}\;\cdot \;r_{ij}\right)}{|r_{ij}{|}^{2}+\frac{1}{100}h^{2}}.$$

#### 2.2.10. SPH Surface Tension

Using the quantities derived in Section 2.2.2 we are able to formulate the expressions for the normal surface tension acceleration in analogy to Equation (5),

(35) $$f_{i}^{n}=-\frac{\delta_{i}}{\rho_{i}}\;\sigma_{n}\;\kappa_{i}\;n_{i},$$

and for the tangential surface tension acceleration in analogy to Equation (6),

(36) $$f_{i}^{t}=\frac{\delta_{i}}{\rho_{i}}\;\sigma_{t}\;\left(\nabla T_{i}-\left(\nabla T_{i}\;\cdot \;n_{i}\right)n_{i}\right).$$

The sum $f_{i}^{S}=f_{i}^{n}+f_{i}^{t}$ enters the SPH momentum balance (Equation (26)).

#### 2.2.11. SPH Wetting

Following [3,8] we use a modification of the surface normal $n_{i}$ of a fluid particle *i* in the vicinity of a wall in order to enforce an equilibrium contact angle. To do so, we first extrapolate the surface normal $n_{j}$ of the wall particles *j* on each fluid particle *i*,

(37) $$n_{i}^{w}=\frac{\sum_{j\in \mathrm{wall}}n_{j}\;W_{ij}\;m_{j}/\rho_{j}}{\left|\sum_{j\in \mathrm{wall}}n_{j}\;W_{ij}\;m_{j}/\rho_{j}\right|}.$$

A corresponding tangent is then evaluated by

(38) $$t_{i}^{w}=\frac{n_{i}-\left(n_{i}\;\cdot \;n_{i}^{w}\right)n_{i}^{w}}{\left|n_{i}-\left(n_{i}\;\cdot \;n_{i}^{w}\right)n_{i}^{w}\right|}.$$

Similarly to Equation (37) the wall contact angle $\theta_{j}$ is mapped onto the fluid particles,

(39) $$\theta_{i}^{w}=\frac{\sum_{j\in \mathrm{wall}}\theta_{j}\;W_{ij}\;m_{j}/\rho_{j}}{\sum_{j\in \mathrm{wall}}W_{ij}\;m_{j}/\rho_{j}}.$$

In many situations the wall contact angle will be a constant but Equation (39) allows to model also graded or sharp variations of the wetting behavior. Next, the distance to the wall is calculated for each fluid particle by

(40) $$d_{i}^{w}=\min_{j\in \mathrm{wall}}r_{ij}\;\cdot \;n_{i}^{w}.$$

This wall distance is used to obtain a normalized weight,

(41) $$w_{i}^{w}=\left\{\begin{array}{cc} 1, & d_{i}^{w}\geq 2h,\\ \frac{d_{i}^{w}-\Delta x}{2h-\Delta x}, & \Delta x<d_{i}^{w}<2h,\\ 0, & d_{i}^{w}\leq \Delta x. \end{array}\right.$$

Finally, the smooth modification is applied,

(42) $$n_{i}^{*}=n_{i}\;w_{i}^{w}+\left(t_{i}^{w}\sin\theta_{i}^{w}+n_{i}^{w}\cos\theta_{i}^{w}\right)\left(1-w_{i}^{w}\right),$$

and $n_{i}^{*}$ is used in Equations (35) and (36) instead of $n_{i}$.

#### 2.2.12. SPH Particle Shifting

A particle shifting technique as introduced by Sun [24] can be used to homogenize the particle distribution. The position shifting for a particle *i* inside the material, i.e., not in the surface region, within a time step Δt is defined as

(43) $$\Delta r_{i}^{\mathrm{PST}}=-0.2\;h\;s\;\Delta t\sum_{j}\left[1+0.2\;{\left(\frac{W_{ij}}{W(\Delta x,h)}\right)}^{4}\right]\frac{2\;m_{j}}{\rho_{i}+\rho_{j}}\nabla W_{ij}.$$

For particles within the surface region and with $\lambda_{i}\geq 0.4$, the position shifting is modified into $\left(I-n_{i}⊗n_{i}\right)\Delta r_{i}^{\mathrm{PST}}$ where I is the identity matrix. In the vicinity of a wall, $n_{i}$ is replaced by $n_{i}^{*}$. For surface region particles with $\lambda_{i}<0.4$, no position shifting is applied.

#### 2.2.13. SPH Time Integration

The numerical integration time step is based on the fastest physical mechanism in the simulation. The competing mechanisms are inertia, sound propagation, surface tension, viscous diffusion and thermal diffusion. Time step conditions leading to stable simulations involving these mechanisms are given in [1,4,39,40,41], respectively,

(44) $$\Delta t=\frac{1}{4}\min\left\{\sqrt{\frac{h}{f_{\max}}},\;\frac{h}{s},\;\sqrt{\frac{\rho_{0}\;h^{3}}{2\pi \;\sigma_{n}}},\;\frac{\rho_{0}\;h^{2}}{2\;\eta },\;\frac{\rho_{0}\;c\;h^{2}}{2\;k}\right\},$$

where $f_{\max}$ is the magnitude of the maximum acceleration of all particles. It is usually suggested to choose the numerical speed of sound *s* about 10 times faster than the maximum particle velocity in the simulation [2]. In our visco-capillary simulations, we found that also the inequality

(45) $$s>3\;\frac{\sigma_{n}}{\eta }$$

should be fulfilled in order to maintain stability.

Time integration is done using a velocity Verlet scheme [42]. The density is propagated by

(46) $$\rho_{i}\left(t+\Delta t\right)=\rho_{i}\left(t\right)+\frac{\Delta t}{2}\left(\frac{D\rho_{i}\left(t\right)}{Dt}+\frac{D\rho_{i}\left(t+\Delta t\right)}{Dt}\right).$$

The specific thermal energy is propagated by

(47) $$H_{i}\left(t+\Delta t\right)=H_{i}\left(t\right)+\frac{\Delta t}{2}\left(\frac{DH_{i}\left(t\right)}{Dt}+\frac{DH_{i}\left(t+\Delta t\right)}{Dt}\right).$$

The velocity is propagated by

(48) $$v_{i}\left(t+\Delta t\right)=v_{i}\left(t\right)+\frac{\Delta t}{2}\left(\frac{Dv_{i}\left(t\right)}{Dt}+\frac{Dv_{i}\left(t+\Delta t\right)}{Dt}\right).$$

Furthermore, the position is propagated including particle shifting by

(49) $$r_{i}\left(t+\Delta t\right)=r_{i}\left(t\right)+\Delta t\;v_{i}\left(t\right)+\frac{\Delta t^{2}}{2}\frac{Dv_{i}\left(t\right)}{Dt}+\Delta r_{i}^{\mathrm{PST}}.$$

## 3. Results

A series of benchmark cases are simulated for which analytical solutions are available for comparison. In Section 3.1, Section 3.2, Section 3.3, Section 3.4, Section 3.5, Section 3.6 and Section 3.7 capillary or thermo-capillary benchmarks are assessed. In Section 3.8, Section 3.9, Section 3.10 and Section 3.11 the free surface thermal boundary conditions are benchmarked. Section 3.12 and Section 3.13 contain more complex cases which provide suggestions for further areas of application of the proposed numerical model.

Unless otherwise stated we are using an ethylene glycol parametrization for the present SPH simulations, i.e., a rest density $\rho_{0}=1113\;kgm{}^{-1}$, a viscosity η=16.1 mPas and a surface tension $\sigma_{n}=48.4\;mNm{}^{-1}$. Ethylene glycol is chosen here as an example for a real fluid which is used in many technical applications. If gravity is used, then g=(0,0,−g) with $g=9.81\;ms{}^{-2}$. Furthermore, the speed of sound is set to $s=10\;ms{}^{-1}$ unless stated otherwise and the isentropic exponent of the equation of state is γ=1. We typically use a ratio of smoothing length and initial particle spacing of h/Δx=1.5 and accordingly $\Xi_{\lambda }=1.88$ for d=2 and $\Xi_{\lambda }=1.82$ for d=3 (compare Appendix A). In Section 3.5 and Section 3.6 the viscosity model according to Equation (30) is used. For all other fluidic benchmarks, the viscosity formulation according to Equation (29) is used. For the benchmark cases in Section 3.5, Section 3.6, Section 3.12 and Section 3.13 artifical viscosity with parameters α=0.01 and β=0.02 is used. For all other cases artificial viscosity is not activated.

All simulations are carried out using the SimPARTIX software developed by Fraunhofer IWM [43].

### 3.1. Droplet Oscillation

As a first benchmark case we analyze the oscillation of a circular droplet with an initial divergence-free velocity field [4,5,6],

(50) $$\begin{array}{ccc} v_{x} & = & v_{0}\frac{x}{r_{0}}\left(1-\frac{y^{2}}{r_{0}r}\right)\exp\left(-\frac{r}{r_{0}}\right), \end{array}$$

(51) $$\begin{array}{ccc} v_{y} & = & -v_{0}\frac{y}{r_{0}}\left(1-\frac{x^{2}}{r_{0}r}\right)\exp\left(-\frac{r}{r_{0}}\right), \end{array}$$

with $v_{0}=1\;ms{}^{-1}$ and $r_{0}=1\;mm$. The lateral and vertical coordinates are *x* and *y*, respectively, and $r=\sqrt{x^{2}+y^{2}}$. The origin is placed at the center of the droplet.

The theoretical oscillation frequency for a 2D droplet is given by

(52) $$f_{0}=\frac{1}{2\pi }\sqrt{\frac{6\;\sigma_{n}}{R^{3}\;\rho_{0}}}.$$

**Figure 1:**
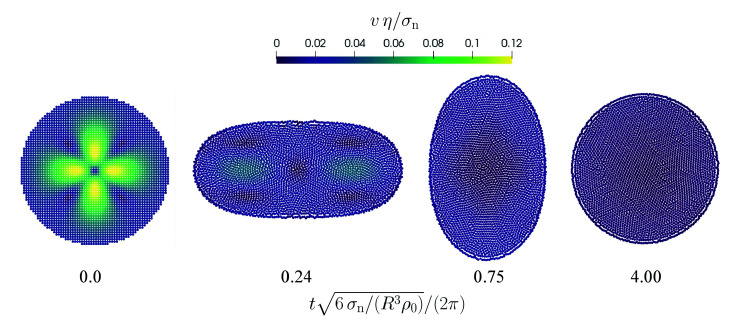
Oscillation of a free droplet with a divergence-free initial velocity field. The normalized velocity magnitude is color-coded. The time is normalized by the oscillation frequency (Equation (52)).

The initial radius of the droplet is R=4 mm and the resolution is varied between Δx=500 μm and Δx=125 μm which corresponds to 8 and 32 particles per radius. Snapshots from the simulation with the finest resolution are shown in Figure 1.

**Figure 2:**
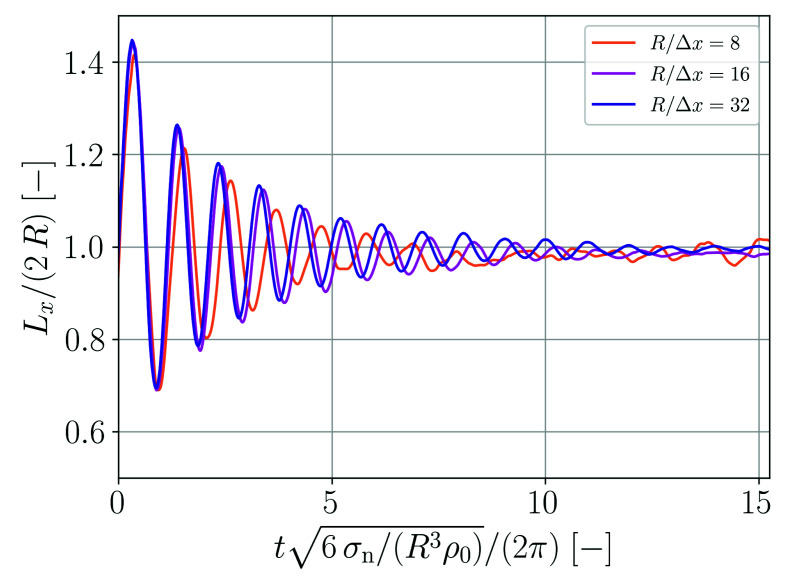
Normalized lateral expansion of the oscillating droplet as a function of normalized time for different spatial resolutions.

The lateral expansion $L_{x}$ of the droplet as a function of time is shown in Figure 2. The regular oscillation with decay in amplitude due to viscous dissipation is clearly perceptible.

**Figure 3:**
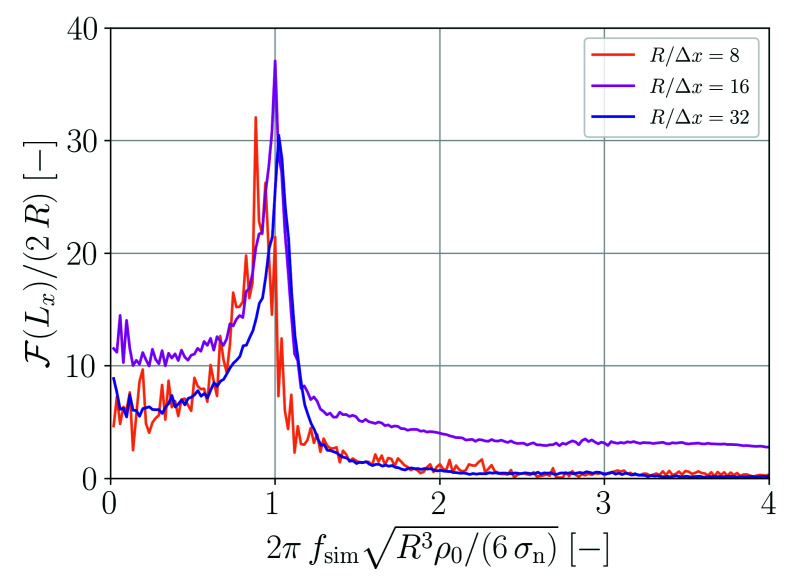
Fourier analysis of the droplet oscillation shown in Figure 2.

A Fourier analysis is carried out in order to assess the oscillation behavior in more detail. The Fourier transform of the droplet expansion, $F(L_{x})$, is shown in Figure 3 as a function of frequency $f_{\mathrm{sim}}$. For the coarsest spatial resolution, the oscillation is too slow. However, for the two finer resolutions the main frequency peak is in good agreement with the theoretical prediction given by Equation (52).

### 3.2. Droplet Formation

**Figure 4:**
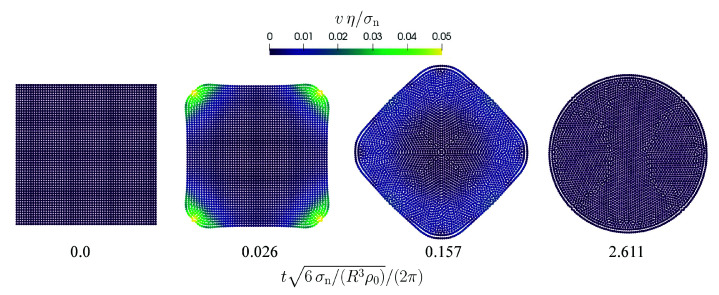
Formation of a droplet from a square under the influence of surface tension. The normalized velocity magnitude is color-coded. The time is normalized using a characteristic oscillation frequency according to Equation (52).

The second benchmark is the formation of a droplet from an initial square arrangement of SPH particles as proposed by Adami et al. [6]. The edge length of the square is L=6mm and resolutions between 15 and 60 particles per edge length are used yielding a particle spacing between Δx=400 μm and Δx=100 μm. Figure 4 shows a sequence of characteristic simulation snapshots using $60^{2}$ particles.

**Figure 5:**
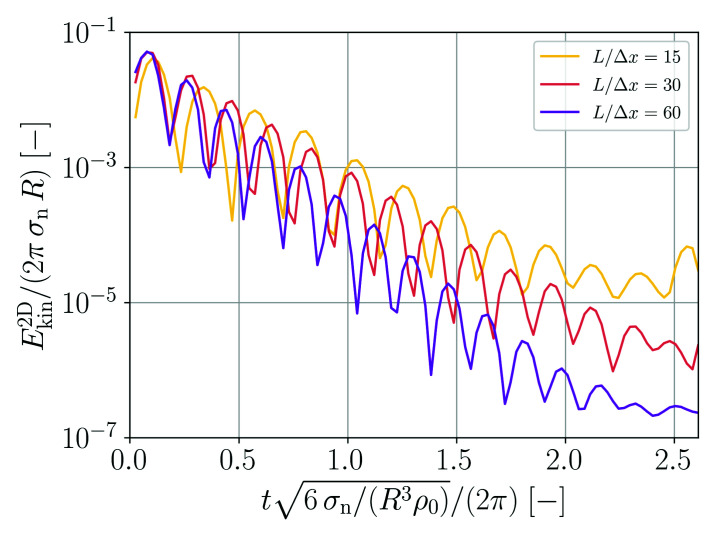
Normalized 2D kinetic energy as a function of normalized time for different spatial resolutions of droplets forming from an initial square patch.

The temporal evolution of the two-dimensional kinetic energy of the droplet for the different spatial resolutions is shown in Figure 5. Following a sharp initial increase of kinetic energy, a decay can be observed for all cases. The decay amounts to about three orders of magnitude for the coarsest resolution while it amounts to more than five orders of magnitude for the finest resolution.

**Figure 6:**
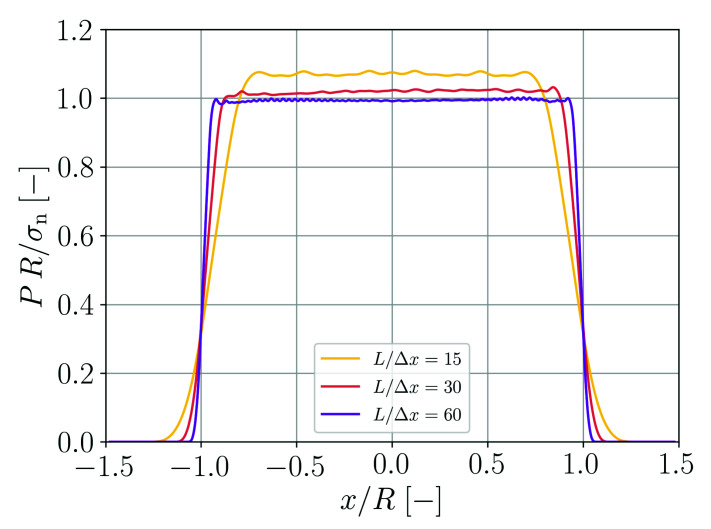
Pressure distribution in the formed droplet as a function of the lateral coordinate at y=0 for different spatial resolutions.

The pressure profile in the eventually formed droplet is evaluated using the SPH approximation according to Equation (9). The results are shown in Figure 6. The finer the spatial resolution the better is the pressure jump at the droplet surface approximated. The agreement with the Young–Laplace equation,

(53) $$P=\sigma_{n}/R=\sigma_{n}\;\sqrt{\pi }/L,$$

which relates the pressure *P* in the droplet to its radius *R*, also gets better for finer spatial resolutions.

**Figure 7:**
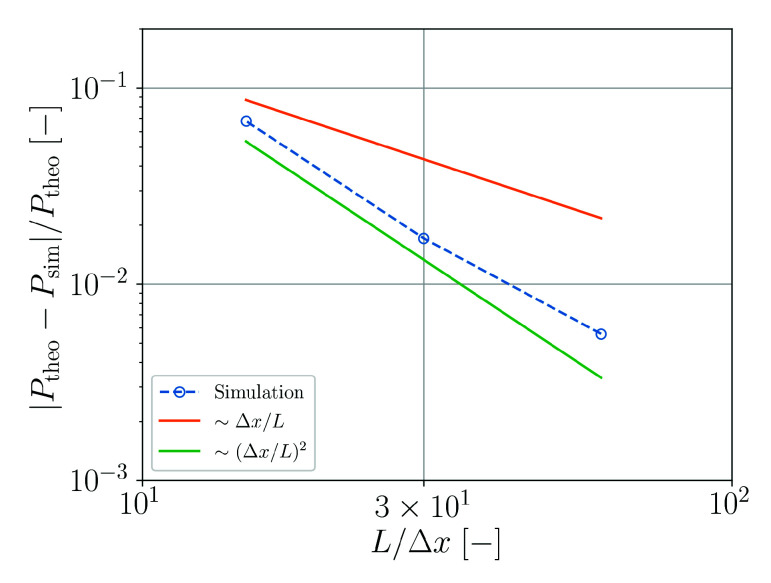
Relative error of the numerically predicted pressure in the droplet as a function of the spatial resolution.

The convergence of the numerical solution $P_{\mathrm{sim}}$ with respect to the theoretical result $P_{\mathrm{theo}}$ for the pressure in the droplet is analyzed in Figure 7. It can be observed that the convergence behavior is significantly better than linear and only slightly worse than quadratic.

**Figure 8:**
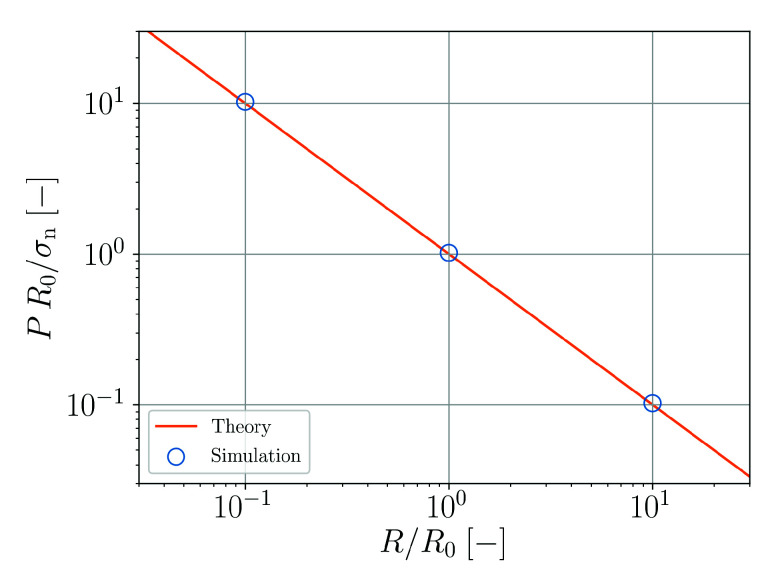
Young–Laplace scaling (Equation (53)) of the normalized droplet pressure as function of the normalized droplet radius.

As a final test, the Young–Laplace scaling with *R* or *L*, respectively, is tested. In this case, *L* is increased by a factor of 10 or decreased by a factor of 0.1 but the particle number of $30^{2}$ is kept constant. The droplet radius of the reference case is referred to as $R_{0}$. Figure 8 shows that the Young–Laplace scaling is well reproduced.

### 3.3. Horizontal Substrate Wetting

**Figure 9:**
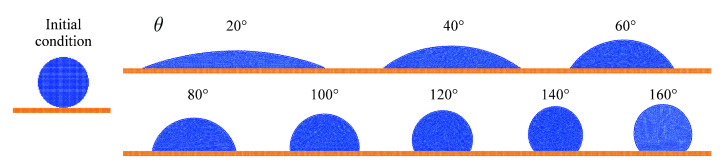
Initial setup and final states of wetting of a substrate for varied numerical contact angle parameter θ.

The third benchmark assesses the wetting behavior of the model similarly to [8]. An initial circular droplet of particles with a radius of R=3 mm is placed directly above a planar substrate. The contact angle parameter is varied between $\theta =20{}^{\circ }$ and $\theta =160{}^{\circ }$. No gravity is applied is order to compare the results with simple analytical solutions. Gravity, of course, would have an effect on a droplet of this size. The resulting droplet shapes on the substrate are shown in Figure 9 for a particle spacing of Δx=100 μm.

The predictive quality of these simulations is analyzed by measuring the equilibrium height $H_{\mathrm{eq}}$ and base diameter $D_{\mathrm{eq}}$ of the resulting droplets and comparing them with the analytical results

(54) $$H_{\mathrm{eq}}=\left(1-\cos\theta \right)\;\sqrt{\frac{\pi \;R^{2}}{\theta -\sin\theta \cos\theta }}$$

and

(55) $$D_{\mathrm{eq}}=2\sin\theta \;\sqrt{\frac{\pi \;R^{2}}{\theta -\sin\theta \cos\theta }}.$$

**Figure 10:**
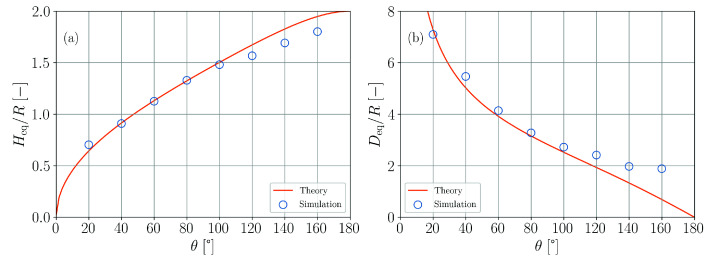
Comparison of simulation results and theory for the wetting of a planar substrate. (**a**) Normalized droplet height as a function of contact angle parameter. (**b**) Normalized droplet base diameter as a function of contact angle parameter.

Figure 10 shows the according simulation results in comparison to the theoretical predictions. The non-trivial shape of both analytical functions is well reproduced up to a contact angle of $\theta =100{}^{\circ }$. Noticeable deviations occur for larger contact angles and the error increases with increasing contact angle.

### 3.4. Vertical Plate Wetting

**Figure 11:**
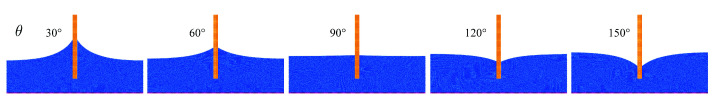
Final states of wetting of a vertical plate for varied numerical contact angle parameter θ.

As fourth benchmark, the wetting of a vertically oriented plate immersed into a fluid is studied. The system is 15 mm wide with periodic boundary conditions in lateral direction. The initial fluid filling height is 4 mm. A vertical plate is immersed into the fluid down to a distance of 1.5 mm from the ground. The acceleration of gravity acts in downward direction and the contact angle is varied between $\theta =30{}^{\circ }$ and $\theta =150{}^{\circ }$. A particle spacing of Δx=25 μm is used. Figure 11 shows simulation snapshots after the terminal height of capillary rise or depression, respectively, is reached.

**Figure 12:**
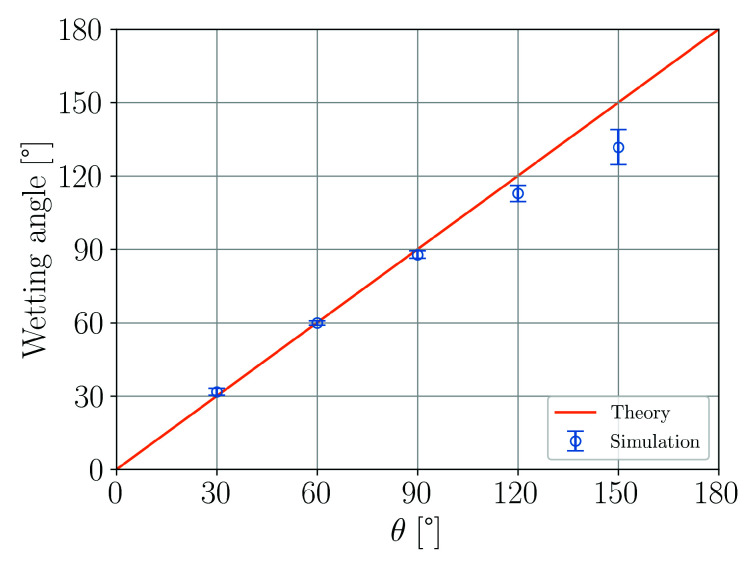
Comparison of simulation results and theory for the wetting of a vertical plate.

The accuracy of the simulations is evaluated by comparing the contact angle θ which enters the numerical model via Equations (39) and (42) with the effective, measured wetting angle with respect to the plate. Figure 12 shows that up to $\theta =90{}^{\circ }$ the agreement is very good. For larger contact angles deviations become more pronounced similarly to the horizontal substrate wetting case in Section 3.3.

### 3.5. Flow between Parallel Plates

**Figure 13:**
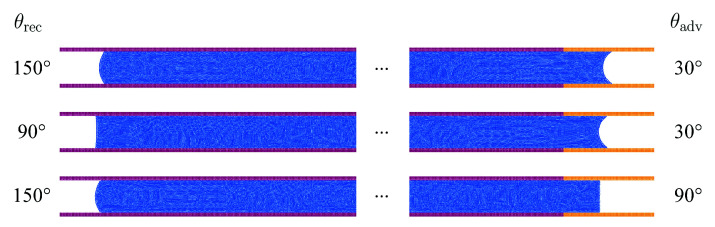
Variation of advancing and receding contact angle for flow between parallel plates.

In the fifth benchmark the steady-state flow of a fluid column between parallel plates is analyzed [26]. The length of the column is $h_{0}=10\;cm$ and distance of the plates is either L=0.5 mm or L=1 mm. The spatial resolution is Δx=25 μm. Different pressure profiles and steady-state velocities of the fluid column are enforced by variation of the advancing contact angle $\theta_{\mathrm{adv}}$ and the receding contact angle $\theta_{\mathrm{rec}}$ shown in Figure 13.

The pressure at the advancing end of the fluid column is given by

(56) $$P_{\mathrm{adv}}=-\frac{2\;\sigma_{n}\;\cos\theta_{\mathrm{adv}}}{L}$$

and at the receding end of the fluid column by

(57) $$P_{\mathrm{rec}}=-\frac{2\;\sigma_{n}\;\cos\theta_{\mathrm{rec}}}{L}.$$

**Figure 14:**
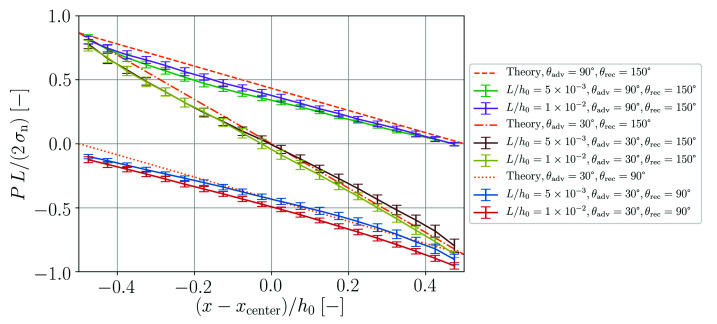
Normalized pressure as a function of normalized position along the fluid column from numerical simulations and according to theory (Equations (56) and (57)).

An overview of the pressure drop along the fluid column for all simulated cases in comparison to the theory is presented in Figure 14. The obtained pressure profiles are generally in agreement with the analytical predictions. Yet, some finite curvature can be observed in the simulation results while the pressure gradient should be constant in theory.

The average steady-state velocity of the fluid in the channel is

(58) $$\langle v\rangle =\frac{L\;\sigma_{n}}{6\;h_{0}\;\eta }\left(\cos\theta_{\mathrm{adv}}-\cos\theta_{\mathrm{rec}}\right).$$

**Figure 15:**
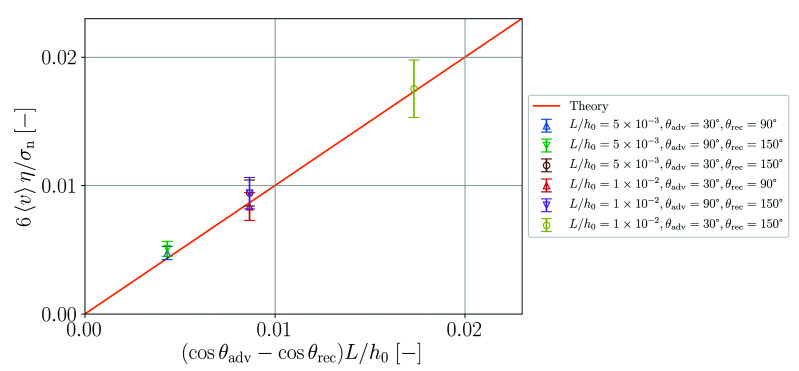
Normalized velocity as function of normalized pressure gradient in the simulations and according to theory (Equation (58)).

This theoretical prediction is compared to the simulation results in Figure 15. Within the error bars of the simulation, which represent one standard deviation, agreement with the theory is obtained. Note that in order to obtain stable simulations for this benchmark case it is necessary to increase the numerical speed of sound to $s=30\;ms{}^{-1}$. For smaller values of *s*, fragmentation of the particle arrangement in the vicinity of the concave surface is observed. This phenomenon is discussed in Section 4.

### 3.6. Flow into a Nozzle

**Figure 16:**
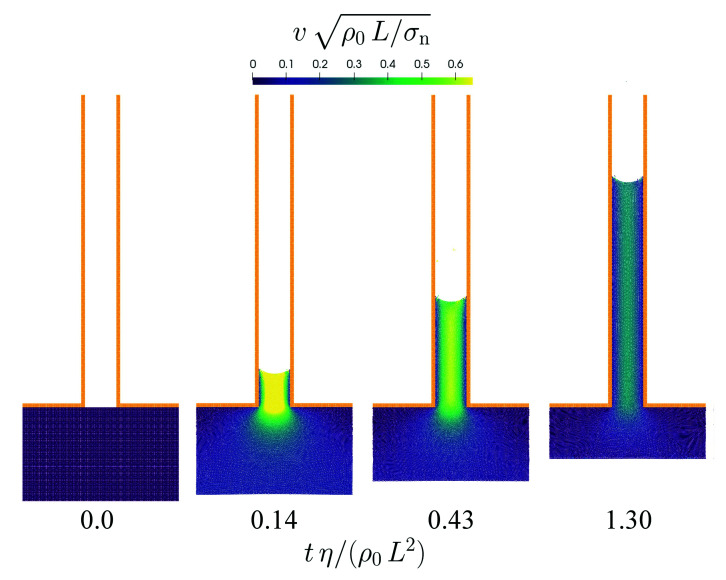
Flow from a reservoir into a nozzle formed by parallel plates. The normalized velocity magnitude is color-coded. The time is normalized using a Reynolds number.

The sixth benchmark is similar to the previous one with the difference that the parallel plates are attached to a fluid reservoir resulting in a nozzle geometry. The distance of the parallel plates is either L=0.5 mm or L=1 mm and the spatial resolution is Δx=25 μm. The capillary action of the surface tension with a contact angle $\theta =30{}^{\circ }$ causes the fluid from the reservoir to penetrate the space between the parallel plates. The rise velocity of the fluid decreases over time as shown in Figure 16.

The rise height $h_{r}$ of the fluid column between the plates and the average rise velocity $v_{r}$ are obtained by solving the set of differential equations [44]

(59) $$\begin{array}{ccc} \frac{d}{dt}\left(h_{r}\;v_{r}\right) & = & -h_{r}\;v_{r}\frac{12\;\eta }{\rho_{0}\;L^{2}}+\frac{2\;\sigma_{n}\;\cos\theta }{\rho_{0}\;L}, \end{array}$$

(60) $$\begin{array}{ccc} \frac{d}{dt}h_{r} & = & v_{r}. \end{array}$$

The according solutions are explicitly given by

(61) $$h_{r}\left(t\right)=\frac{\sqrt{L^{3}\;\rho_{0}\;\sigma_{n}\;\cos\theta }}{6\;\eta }\sqrt{\frac{12\;\eta \;t}{\rho_{0}\;L^{2}}+\exp\left(-\frac{12\;\eta \;t}{\rho_{0}\;L^{2}}\right)-1}$$

and

(62) $$v_{r}\left(t\right)=\sqrt{\frac{\sigma_{n}\;\cos\theta }{\rho_{0}\;L}}\frac{1-\exp\left(-\frac{12\;\eta \;t}{\rho_{0}\;L^{2}}\right)}{\sqrt{\frac{12\;\eta \;t}{\rho_{0}\;L^{2}}+\exp\left(-\frac{12\;\eta \;t}{\rho_{0}\;L^{2}}\right)-1}}.$$

**Figure 17:**
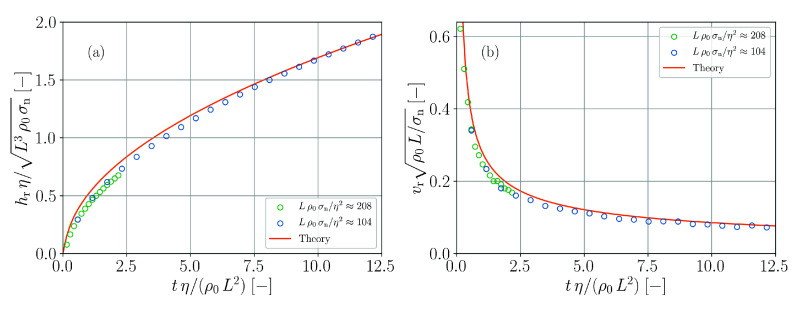
Flow into a nozzle. Normalized rise height (**a**) and normalized rise velocity (**b**) as functions of normalized time in simulations and according to theory (Equations (61) and (62)).

These theoretical predictions are compared with the data obtained from the numerical simulations in Figure 17. The transient behavior of both height and velocity are satisfactorily reproduced in the simulations although the actual values are systematically underestimated to a small extent. Note that in order to obtain stable simulations for this benchmark case it is necessary to increase the numerical speed of sound to $s=50\;ms{}^{-1}$. The reason for this is the avoidance of fragmentation close to the surface similarly to the case in Section 3.5. The even higher speed of sound in this case is required because of the strong acceleration during the initial phase of the flow into the nozzle.

### 3.7. Marangoni Flow

The seventh benchmark focuses on the effect of a surface tension gradient caused by a temperature gradient [9]. A fluid initially rests in a basin of L=10 mm width. The left wall of the basin has a constant temperature of $T_{\min}=300\;K$ and the right wall a constant temperature of $T_{\max}=T_{\min}+\Delta T=400\;K$. The fluid temperature initially describes a linear profile interpolating between the values of the walls. The basin bottom is adiabatic. The specific heat capacity of the fluid is $c=1000\;Jkg{}^{-1}K{}^{-1}$, the thermal conductivity is $k=10\;Wm{}^{-1}K{}^{-1}$ and $\sigma_{t}=-1\;mNm{}^{-1}K{}^{-1}$. The particle spacing is varied between Δx=100 μm and Δx=400 μm. The contact angle with respect to both side walls is $\theta =90{}^{\circ }$.

**Figure 18:**
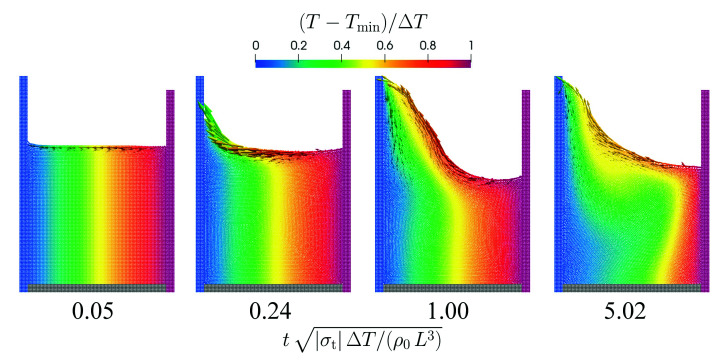
Flow in a basin driven by Marangoni surface current. The normalized temperature is color-coded and the size of the velocity vectors is proportional to the velocity magnitude. The time is normalized by a characteristic Marangoni time scale.

Images from the simulation using the spatial resolution L/Δx=100 are displayed in Figure 18. The fluid strongly rises on the left wall due to the Marangoni effect which drives the fluid at the surface from the warm region towards the cold region. The last snapshot shows the steady state where an eddy has formed below the surface.

**Figure 19:**
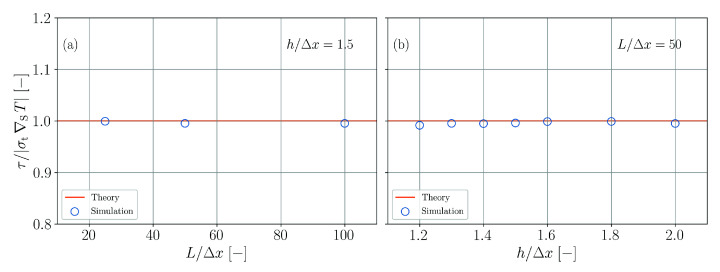
Tangential surface tension induced by temperature gradient. Normalized tension as a function of the spatial resolution (**a**) and as a function of the normalized smoothing length (**b**).

The model is quantitatively assessed by evaluating the Marangoni tension at the surface at the beginning of the simulation using different spatial resolutions as well as different ratios of smoothing length and particle spacing. For this purpose, the tangential volumetric surface force (Equation (6)) is evaluated using SPH interpolation and then integrated along a line perpendicular to the surface. The result τ should be equal to $|\sigma_{t}\;\nabla_{S}T|=10\;\mathrm{Pa}$. Figure 19 shows that the theoretical result is met for all studied resolutions and smoothing length ratios.

### 3.8. Plate with Double-Sided Heat Transfer

The eighth to eleventh benchmark case do not involve any fluid flow but assess the predictive quality of the thermal boundary conditions at the surface as introduced in Section 2.2.4. A convenient parameter for these cases is the thermal diffusivity, $a=k/(\rho_{0}\;c)$, as it allows to normalize the time and a characteristic length scale in terms of a Fourier number. In all of these four cases the thermal conductivity is $k=1\;Wm{}^{-1}K{}^{-1}$, the specific heat capacity is $c=100\;Jkg{}^{-1}K{}^{-1}$ and the density is $\rho_{0}=7800\;kgm^{-3}$.

As eighth benchmark an infinite plate with heat transfer on both surfaces is analyzed. The plate thickness is 2L=2 mm and its initial temperature is $T_{0}=300\;K$. The heat transfer coefficient is $f=1\times 10^{4}\;Wm^{-2}K{}^{-1}$ and the ambient temperature is $T_{a}=400\;K$.

The analytical solution for this case is given by the series

(63) $$\frac{T\left(x,t\right)-T_{0}}{T_{a}-T_{0}}=1-2\sum_{n=1}^{\infty }\frac{\sin\left(\gamma_{n}\right)\cos\left(\gamma_{n}\;x/L\right)\exp\left(-\gamma_{n}^{2}\;a\;t/L^{2}\right)}{\gamma_{n}+\sin\left(\gamma_{n}\right)\cos\left(\gamma_{n}\right)},$$

where $\gamma_{n}$ is the *n*th root of the implicit equation

(64) $$\frac{f\;L}{k}=\gamma_{n}\tan\left(\gamma_{n}\right).$$

**Figure 20:**
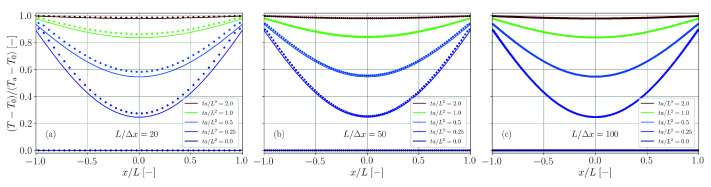
Comparison of simulation results (dots) and analytical solution (solid lines, Equation (63)) for the temperature distribution in a plate with double-sided heat transfer. The spatial resolution is varied between L/Δx=20 (**a**), L/Δx=50 (**b**) and L/Δx=100 (**c**).

The solution is calculated up to $n_{\max}=500$ and compared with the simulation results for different spatial resolutions in Figure 20. While for the lowest spatial resolution the transient temperature is overestimated, differences between theory and simulation vanish for a sufficiently fine resolution.

### 3.9. Plate with Double-Sided Heat Flux

The ninth benchmark is similar to the eighth one with the difference that instead of heat transfer a heat flux $q=1\times 10^{4}\;Wm^{-2}$ is prescribed at both surfaces of the plate.

The solution for this situation is given by

(65) $$\frac{(T\left(x,t\right)-T_{0})\;k}{q\;L}=\frac{a\;t}{L^{2}}+\frac{x^{2}}{2\;L^{2}}-\frac{1}{6}-2\sum_{n=1}^{\infty }\frac{{\left(-1\right)}^{n}}{\gamma_{n}^{2}}\cos\left(\gamma_{n}\;x/L\right)\exp\left(-\gamma_{n}^{2}\;a\;t/L^{2}\right),$$

with $\gamma_{n}$ defined by

(66) $$\gamma_{n}=n\;\pi .$$

**Figure 21:**
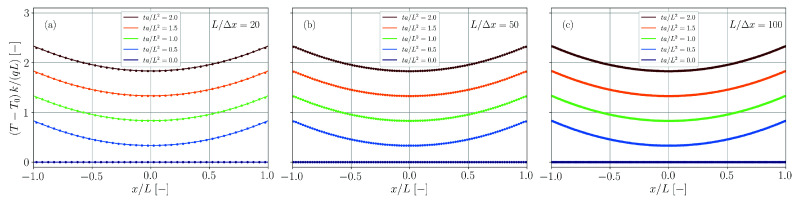
Comparison of simulation results (dots) and analytical solution (solid lines, Equation (65)) for the temperature distribution in a plate with double-sided heat flux. The spatial resolution is varied between L/Δx=20 (**a**), L/Δx=50 (**b**) and L/Δx=100 (**c**).

Again, the series is evaluated up to $n_{\max}=500$ and simulation and theory are compared in Figure 21 for different spatial resolutions. Perfect agreement is found independent of the actual resolution.

### 3.10. Plate with Heat Transfer and Heat Flux

The tenth benchmark is a combination of the two previous ones. An infinite plate of thickness L=1mm experiences heat transfer with $f=1\times 10^{4}\;Wm^{-2}K{}^{-1}$ and $T_{a}=300\;K$ at x/L=0 and heat flux with $q=1\times 10^{4}\;Wm^{-2}$ at x/L=1. The initial temperature of the plate is $T_{0}=T_{a}$.

The analytical series solution is given by

(67) $$\frac{(T\left(x,t\right)-T_{0})\;f}{q}=1+\frac{f\;x}{k}-\sum_{n=1}^{\infty }\left(\cos\left(\gamma_{n}\;x/L\right)+\frac{f\;L}{k\;\gamma_{n}}\sin\left(\gamma_{n}\;x/L\right)\right)\frac{2\;\sin\left(\gamma_{n}\right)\exp\left(-\gamma_{n}^{2}\;a\;t/L^{2}\right)}{\gamma_{n}+\sin\left(\gamma_{n}\right)\cos\left(\gamma_{n}\right)},$$

with the roots $\gamma_{n}$ given by

(68) $$\frac{f\;L}{k}=\gamma_{n}\tan\left(\gamma_{n}\right).$$

**Figure 22:**
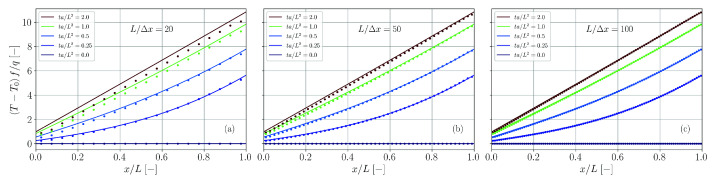
Comparison of simulation results (dots) and analytical solution (solid lines, Equation (67)) for the temperature distribution in a plate with heat transfer and heat flux. The spatial resolution is varied between L/Δx=20 (**a**), L/Δx=50 (**b**) and L/Δx=100 (**c**).

A comparison between simulation and theory ($n_{\max}=500$) for different spatial resolutions is provided in Figure 22. For too coarse resolutions and Fourier numbers $ta/L^{2}≳1$ the temperature is underestimated by the simulation. Yet, similarly to the benchmark case with double-sided heat transfer (Section 3.8), the analytical solution gets better approximated with finer spatial resolution.

### 3.11. Rod with Heat Flux

In the eleventh benchmark an infinite rod of radius R=1 mm and initial temperature $T_{0}=300\;K$ is heated by the surface heat flux $q=1\times 10^{4}\;Wm^{-2}$.

The according analytical solution for the temperature distribution along the radial coordinate *r* is given by

(69) $$\frac{(T\left(r,t\right)-T_{0})\;k}{q\;R}=\frac{2\;a\;t}{R^{2}}+\frac{r^{2}}{2\;R^{2}}-\frac{1}{4}-2\sum_{n=1}^{\infty }\frac{J_{0}\left(\gamma_{n}\;r/R\right)\exp\left(-\gamma_{n}^{2}\;a\;t/R^{2}\right)}{\gamma_{n}^{2}\;J_{0}\left(\gamma_{n}\right)},$$

with the *n*th root $\gamma_{n}$ given by

(70) $$J_{1}\left(\gamma_{n}\right)=0.$$

**Figure 23:**
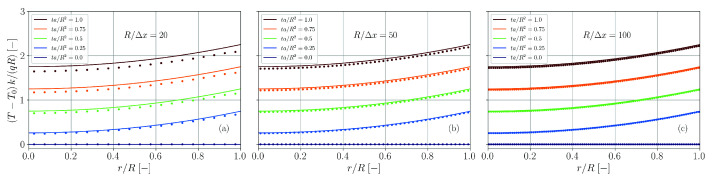
Comparison of simulation results (dots) and analytical solution (solid lines, Equation (69)) for the temperature distribution in a rod with surface heat flux. The spatial resolution is varied between R/Δx=20 (**a**), R/Δx=50 (**b**) and R/Δx=100 (**c**).

Due to the rotational symmetry of this benchmark case, the Bessel functions of the first kind $J_{0}$ and $J_{1}$ appear in the solution. The comparison between the simulation results and the analytical solution in Figure 23 reveals that the temperature is underestimated for too coarse spatial resolutions. However, for finer resolution the analytical result is well predicted.

**Figure 24:**
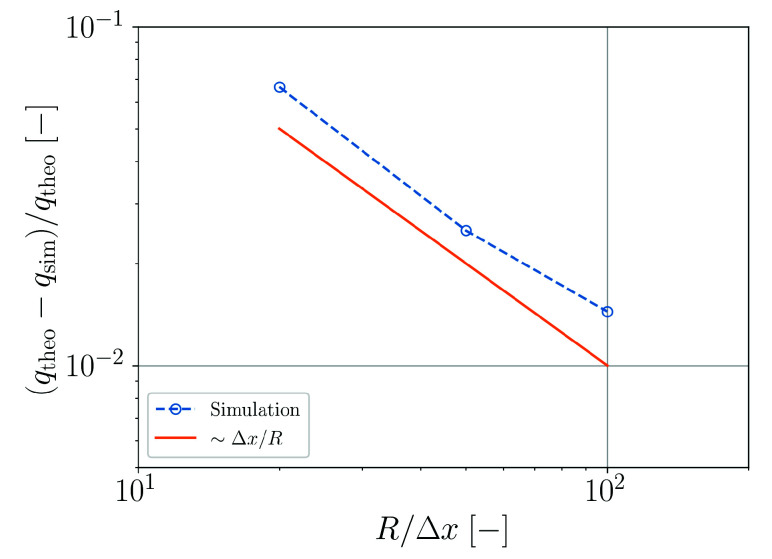
Relative error of the numerically predicted heat flux in a rod as a function of the spatial resolution.

For this case, the accuracy of the surface heat transfer in the simulation is assessed by evaluating the rate of change of thermal energy in the simulation. As heat transfer is the only active heating mechanism, the rate of thermal energy change should be directly proportional to the applied heat flux. An according convergence analysis is plotted in Figure 24. The effective heat flux in the simulation converges approximately linear with the spatial resolution.

### 3.12. Droplet Deformation and Splitting

**Figure 25:**
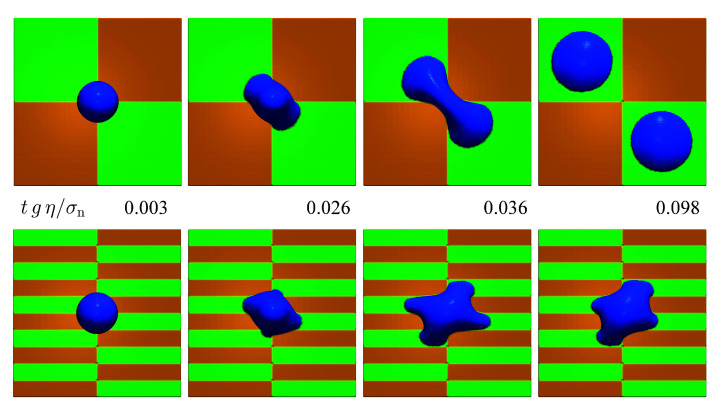
Wetting behavior of a droplet on substrates composed of hydrophilic regions (green, $\theta =45{}^{\circ }$) and hydrophobic regions (orange, $\theta =135{}^{\circ }$).

The twelfth case is intended to study the usage of the presented model in a more complex three-dimensional situation. A droplet of fluid with a diameter of 1 mm is discretized using Δx=20 μm. The droplet floats above a substrate initially. Gravity pushes the droplet onto the substrate which consists of hydrophilic regions with a contact angle of $\theta =45{}^{\circ }$ and hydrophobic regions with a contact angle of $\theta =135{}^{\circ }$. Two different substrates are used. One is composed of square regions having a certain contact angle. The other substrate consists of striped regions with a width of 400 μm. The numerical speed of sound in the simulation is $s=3\;ms{}^{-1}$. Figure 25 shows the temporal evolution of the fluid droplet on both substrates. For the square regions, the wetting and de-wetting behavior leads eventually to a splitting of the droplet into two. For the striped regions, the droplet does not split up but ends up with a very peculiar shape of its perimeter.

### 3.13. Thermo-Visco-Capillary Droplet Coalescence

**Figure 26:**
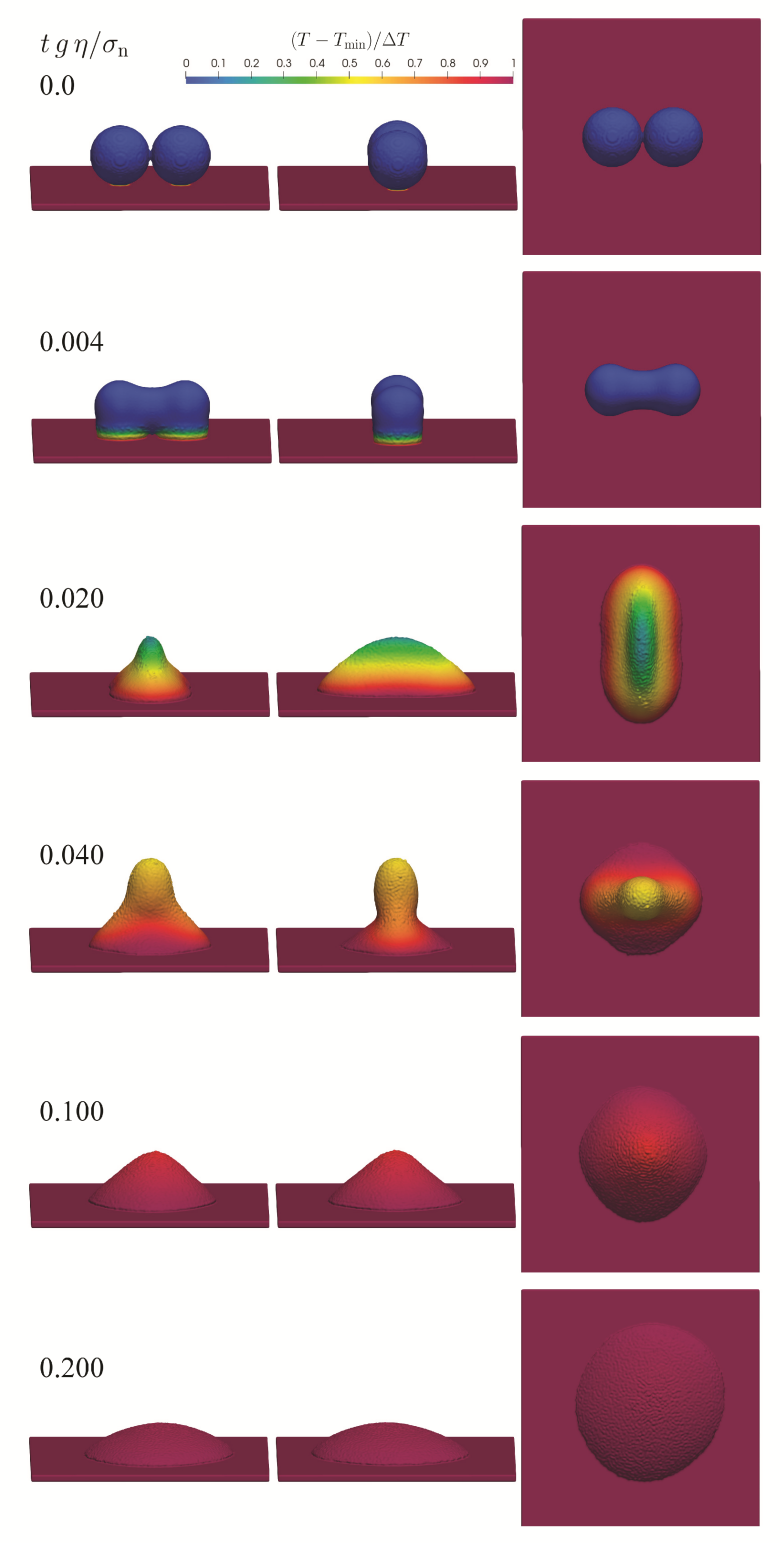
Coalescence of two droplets under the influences of temperature-dependent viscosity and surface tension.

The thirteenth and final case contains the most complex simulation setup. Two droplets of fluid with diameters of 1 mm are discretized using Δx=20 μm. The droplets touch a substrate below as well as each other initially. Gravity acts normal to the substrate surface. The fluid has an initial temperature of $T_{\min}=300\;K$ and the substrate a constant temperature of $T_{\max}=T_{\min}+\Delta T=500\;K$. The specific heat capacity of the fluid is $c=1000\;Jkg{}^{-1}K{}^{-1}$ and the thermal conductivity of both fluid and substrate is $k=10\;Wm{}^{-1}K{}^{-1}$. The surface tension of the fluid is

(71) $$\sigma_{n}\left(T\right)={\tilde{\sigma }}_{n}+\sigma_{t}\left(T-T_{\min}\right)$$

with ${\tilde{\sigma }}_{n}=48.4\;mNm{}^{-1}$ and $\sigma_{t}=-0.2\;mNm{}^{-1}K{}^{-1}$. The contact angle with respect to the substrate is $\theta =45{}^{\circ }$. The ambient temperature is $T_{a}=T_{\min}$ and the fluid is cooled by surface heat transfer with $f=1\times 10^{2}\;Wm{}^{-2}K{}^{-1}$ and by radiation with ε=0.5. The viscosity of the fluid is temperature-dependent in the form of an Arrhenius relation,

(72) $$\eta \left(T\right)=\tilde{\eta }\exp\left(\frac{E_{a}}{R}\left(\frac{1}{T}-\frac{1}{T_{\min}}\right)\right),$$

with $\tilde{\eta }=16.1\;m\mathrm{Pa}s$, the activation energy $E_{a}=1\times 10^{4}\;J\mathrm{mol}{}^{-1}$ and the universal gas constant $R=8.314\;J\mathrm{mol}{}^{-1}K{}^{-1}$. The numerical speed of sound is $s=3\;ms{}^{-1}$. The thermo-visco-capillary coalescence behavior of the droplets is shown from different perspectives in Figure 26.

## 4. Discussion

The presented consistent modeling of thermo-capillary effects and thermal boundary conditions within the SPH method could be verified and validated by the extensive analyses in Section 3. In the following, we would like to address some details which could be highlighted by the analyses.

For free droplets, the visco-capillary oscillation frequency and the Laplace pressure are adequately reproduced by the simulations with sufficient spatial resolution. For the Laplace pressure, a near quadratic convergence with spatial resolution has been found. At the finest resolution used, the relative error is less than $10^{-2}$, which is typically an acceptable tolerance for SPH simulations [2].

The investigations on static wetting have shown that contact angles up to $\theta \approx 100{}^{\circ }$ are well reproduced in the simulation. Larger contact angles are increasingly poorly reproduced. A comparable observation was made by Breinlinger et al. [8]. This is apparently a systematic weakness of the submodel for imprinting the desired contact angle in Section 2.2.11. The reason for this is that in the approach used to determine the free surface and the quantities derived from it, the fluid and the substrate are initially considered as a common phase. As a result, it is not possible to identify free surfaces that are only separated by small gaps. However, precisely this situation exists in the case of large contact angles. Alternatively, the fluid and the substrate could be considered as distinct phases from the beginning. In that case, however, the reproduction of small contact angles would become increasingly inaccurate, which has been observed in corresponding tests.

In the dynamic wetting scenarios, a satisfactory overall agreement of the simulation with theoretical predictions for the pressure and velocity has been observed. A slight, systematic deviation in the dynamic wetting case in Section 3.6 can be explained by the fact that the momentum of the reservoir is not considered in the respective balance equation (Equation (59)). The strong tensile stresses caused by the surface tension in the dynamic wetting scenarios are seen as the reason why it is necessary to slightly increase the numerical speed of sound in these cases. Without such an increase, fragmentation of the particles near the surface has been observed. In this context, the used approach of a surface delta function based on the minimum eigenvalue λ of the inverse renormalization tensor has proved to be helpful. As shown in the Appendix A, the surface delta function is more evenly distributed among the particle layers on the surface than when using a delta function which is based only on the kernel gradient. This attenuates the tensile forces acting on the individual particles, which helps to stabilize the simulation. The use of a weak artificial viscosity and the viscosity formulation of Sigalotti et al. [38] have also proved to stabilize the dynamic wetting scenarios.

A fundamental strength of the presented model is that only one phase has to be modeled. This saves computational time, since especially for systems consisting of a fluid and a gas, the low density of the gas requires a very small time step, as shown by Colagrossi and Landrini [45]. However, even the presented model may require a relatively small time step due to a high numerical speed of sound, as described in the previous paragraph. For such a situation, the use of an implicit SPH formulation can be useful, such as the ones used by Nair and Pöschel [31] or Fürstenau et al. [20] in the context of capillary phenomena.

The investigations on the heat transfer boundary condition have shown that agreements with analytical results are obtained by simulations with sufficiently fine spatial resolution. In the case of a pure heat flux boundary condition on a flat surface, excellent accuracy of the numerical simulations has been found regardless of the spatial resolution. This can probably be attributed to the fact that the heat input is independent of the temperature computed in the numerical simulation. In the case of a curved surface, a linear convergence of the relative error of the heat flux with the spatial discretization could be determined. This convergence behavior can be considered a measure of how well the surface delta function describes a curved surface.

The two cases in Section 3.12 and Section 3.13 are not intended to validate the model but to provide ideas for application in more complex scenarios. Use cases for thermo-capillary SPH simulations offer, for example, welding processes (Hu and Eberhard [46]) or additive manufacturing processes (Russell et al. [47], Fürstenau et al. [48], Meier et al. [49]). The presented method has already been applied by the author and coworkers to derive universal process maps for laser powder bed fusion of polymers [50].

Based on the identified weakness of the presented model, a more accurate description of large contact angles is considered as a future field of development. In addition, the modeling of dynamic wetting phenomena can be improved by considering the dynamic contact angle as in Huber et al. [11]. A fundamental problem for single-phase models based on the CSF approach is the lack of possibility to formulate the surface tension forces in a momentum-preserving way. As a consequence, sooner or later a non-physical drift of free-floating droplets or of droplets on flat substrates occurs in the simulations. A solution to this problem would be highly desirable in terms of a more universal applicability of the method.

## 5. Conclusions

The main contributions of the present work are summarized in the following list:

- The surface delta function, surface normal and surface curvature are derived in a consistent manner from the renormalization tensor in single-phase SPH simulations.
- Thermo-capillarity, wetting and thermal boundary conditions are formulated in a uniform way based on these surface properties.
- The quantitative agreement of corresponding simulations with a large number of analytical test cases demonstrates the validity of the presented approach.

## Figures and Tables

**Figure A1:**
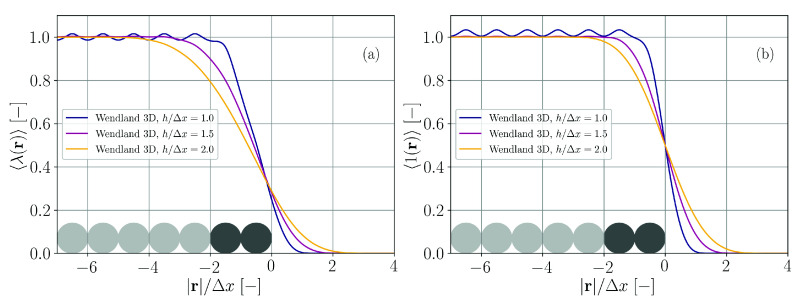
Functions that are approximately 1 inside and 0 outside the particle distribution for different ratios of smoothing length and particle spacing. The 3D Wendland kernel is used as an example. The particle positions are indicated by the circles. (**a**) Minimum eigenvalue of the inverse renormalization tensor (Equation (A1)). (**b**) Unity field (Equation (A2)).

**Figure A2:**
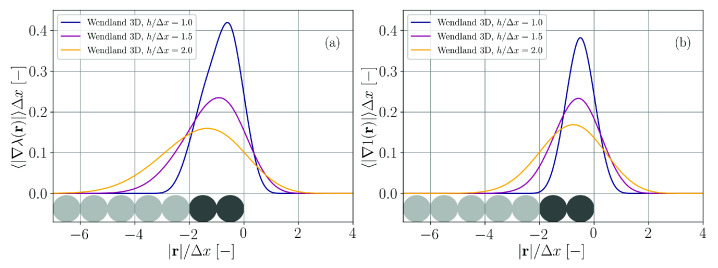
Normalized gradients of the functions from Figure A1. (**a**) Based on the minimum eigenvalue of the inverse renormalization tensor. (**b**) Based on the unity field.

**Figure A3:**
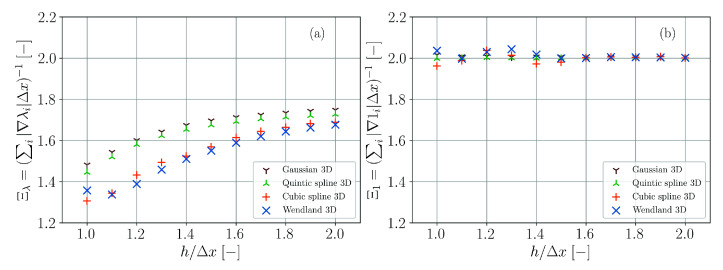
Normalization factors for the surface delta functions for different 3D kernels. (**a**) Based on the minimum eigenvalue of the inverse renormalization tensor. (**b**) Based on the unity field.

**Figure A4:**
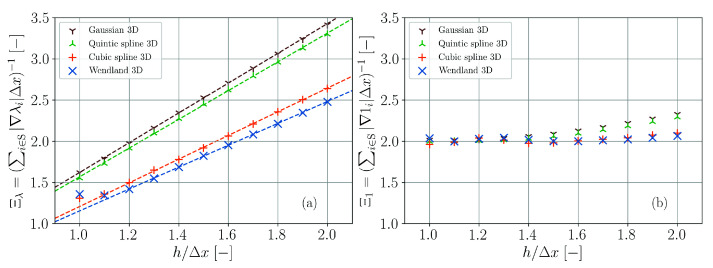
Normalization factors for the surface delta functions for different 3D kernels if the surface calculations are restricted to two particle layers. (**a**) Based on the minimum eigenvalue of the inverse renormalization tensor. (**b**) Based on the unity field.

## Data Availability

The data presented in this study are available on request from the corresponding author.

## Appendix A. Surface Delta Function

Different approaches exist in order to evaluate a suitable surface delta function. Equations (19) and (20) are two possibilities for this purpose. We consider using Equation (19) to be a consistent choice, because this way the surface normal (Equation (18)) and the surface delta function are derived from the same quantity, namely λ. However, as other authors [14,20,23] use Equation (20) to obtain the surface delta function, we compare these two approaches in the following.

The half space x<0 is filled with SPH particles at positions $x_{n}=\left(0.5-n\right)\Delta x,n\in N$. In Figure A1 the SPH interpolations of λ,

(A1) $$\langle \lambda \left(r\right)\rangle =\sum_{j}\frac{m_{j}}{\rho_{j}}\;\lambda_{j}\;W(|r-r_{j}\left|,h\right),$$

and of unity,

(A2) $$\langle 1\left(r\right)\rangle =\sum_{j}\frac{m_{j}}{\rho_{j}}\;W(|r-r_{j}\left|,h\right),$$

are plotted as functions of a coordinate |r| perpendicular to the surface for different ratios of smoothing length and particle spacing. Both for λ and unity the functions drop from 1 to 0 in the vicinity of the surface. Yet, there are subtle differences. The descent appears to be steeper for the case of unity. Furthermore, the inflection point is located exactly at |r|=0 for the unity field while it is shifted somewhat towards the half space filled with particle for the λ field.

The functional forms of the corresponding gradients are obtained by applying Equation (9) to either $|\nabla \lambda_{i}|$ defined by Equation (17) or to $|\nabla 1_{i}|$ defined by Equation (20). The results are plotted in normalized form in Figure A2. The overall shape of the gradients for both fields is similar. While the gradients are symmetric for the unity field, they are a bit skewed for the λ field. In addition, finite values for the gradients are more localized at the surface for the unity field. As to be expected, the smaller the ratio h/Δx the more localized are the gradients of both fields.

The skewedness in case of the λ field is another reason why we prefer it over the unity field. Due to this property the magnitude of the delta function is more evenly distributed over the two layers of surface region particles. The delta function of the unity field is more localized on the outermost particle layer. We found that it is beneficial for the stability of the numerical simulations if the action of the surface tension is more evenly distributed over two particle layers.

In order to obtain the normalization factors $\Xi_{\lambda }$ in Equation (19) and $\Xi_{1}$ in Equation (20) the gradient magnitudes $|\nabla \lambda_{i}|$ and $|\nabla 1_{i}|$ are summed over a column of particles *i* perpendicular to the surface similar to the in [23]. The inverse sums yield the normalization factors shown in Figure A3 for four different kinds of SPH kernel functions. For the case of the λ field, the normalization factor increases with the ratio h/Δx and appears to reach a plateau for large ratios. For the case of the unity field, the normalization factor oscillates around 2 for ratios h/Δx<1.5 and is close to 2 for larger ratios.

As mentioned in Section 2.2.2, we restrict all surface related calculations to those particles which are no more than 1.5Δx away from the free surface particles. Consequently, the normalization factors must reflect this restriction. In Figure A4 the corresponding results are presented. The normalization factors are in good approximation linear functions of the ratio h/Δx for the case of the λ field. For the unity field the normalization factors deviate systematically from 2 and increase with h/Δx if this ratio is larger than ∼1.4.

The linear function to approximate the normalization factor for the λ field as plotted in Figure A4 is given by

(A3) $$\Xi_{\lambda }\left(h/\Delta x\right)=a_{\lambda }\frac{h}{\Delta x}+b_{\lambda }.$$

The slope and intercept values for different SPH kernel functions in two and three dimensions are listed in Table A1.

**Table A1:** Parameters for the $\Xi_{\lambda }$ approximation function (Equation (A3)).

	2D		3D	
**Kernel Function**	$a_{\lambda }$	$b_{\lambda }$	$a_{\lambda }$	$b_{\lambda }$
Wendland (Equation (10))	1.324	−0.108	1.328	−0.174
Cubic spline (Equation (A4))	1.504	−0.273	1.437	−0.231
Quintic spline (Equation (A5))	1.771	−0.173	1.744	−0.177
Gaussian (Equation (A6))	1.805	−0.185	1.807	−0.187

The cubic spline (M4) with ν=5/(14π) for d=2 and ν=1/(4π) for d=3 is defined by

(A4) $$W\left(r,h\right)=\frac{\nu }{h^{d}}\left\{\begin{array}{cc} {\left(2-\zeta \right)}^{3}-4{\left(1-\zeta \right)}^{3}, & 0\leq \zeta <1,\\ {\left(2-\zeta \right)}^{3}, & 1\leq \zeta <2,\\ 0, & \zeta \geq 2. \end{array}\right.$$

The quintic spline (M6) with ν=7/(478π) for d=2 and ν=1/(120π) for d=3 is defined by

(A5) $$W\left(r,h\right)=\frac{\nu }{h^{d}}\left\{\begin{array}{cc} {\left(3-\zeta \right)}^{5}-6{\left(2-\zeta \right)}^{5}+15{\left(1-\zeta \right)}^{5}, & 0\leq \zeta <1,\\ {\left(3-\zeta \right)}^{5}-6{\left(2-\zeta \right)}^{5}, & 1\leq \zeta <2,\\ {\left(3-\zeta \right)}^{5}, & 2\leq \zeta <3,\\ 0, & \zeta \geq 3. \end{array}\right.$$

The Gaussian kernel function with ν=1/π for d=2 and $\nu =1/\pi^{3/2}$ for d=3 is defined by

(A6) $$W\left(r,h\right)=\frac{\nu }{h^{d}}\left\{\begin{array}{cc} \exp(-\zeta^{2}), & 0\leq \zeta <3,\\ 0, & \zeta \geq 3. \end{array}\right.$$
